# Highly cross-linked polyethylene still outperforms conventional polyethylene in THA: 10-year RSA results

**DOI:** 10.1080/17453674.2021.1932140

**Published:** 2021-06-18

**Authors:** Halldor Bergvinsson, Vasilis Zampelis, Martin Sundberg, Gunnar Flivik

**Affiliations:** Department of Orthopedics, Skåne University Hospital, Clinical Sciences, Lund University, Lund, Sweden

## Abstract

Background and purpose — Cup wear in total hip arthroplasty (THA) can be affected by different manufacturing processes of the polyethylene (PE). We report the long-term wear pattern differences, as well as early creep behavior, between conventional PE and highly cross-linked PE (HXLPE) liners, as measured with radiostereometry (RSA) up to 10 years. We also compare migration and clinical outcome of 2 similar uncemented cups with different backside surface roughness.

Patients and methods — We included 45 patients with primary osteoarthritis. 23 received a conventional liner and 22 an HXLPE liner in a similar uncemented cup, but with a slightly rougher surface. The patients were followed up with RSA and hip-specific outcome questionnaire (HOOS) at 3 months, 1, 2, 5, and 10 years.

Results — During the first 3 months both liners showed expected deformation with mean proximal head penetration of 0.39 mm (conventional PE) and 0.21 mm (HXLPE). Between 3 months and 10 years there was a difference in annual wear with 0.12 mm/year for the conventional liner and 0.02 mm/year for the HXLPE liner. The cup with rougher surface had less initial migration but both types had stabilized after 3 months. The HOOS scores improved after surgery and remained high for both groups throughout the study period.

Interpretation — Up to 10 years the HXLPE has consistent lower annual wear, possibly contributing to longer survival of the THA, compared with conventional PE. All patients reported good results regardless of liner type.

Osteolysis, attributed to polyethylene wear debris, is one of the main causes of aseptic loosening in THA (Jacobs et al. 2001). Since highly cross-linked polyethylene (HXLPE) was introduced, several studies have shown reduced wear compared with ultra-high-molecular-weight polyethylene (UHMWPE), hereafter called conventional PE (Kuzyk et al. 2011, van Loon et al. 2020). Conventional PE liners demonstrate a mean wear rate of around 0.1 mm/year, which has been considered as the generally accepted osteolysis threshold. However, according to Dumbleton et al. (2002), a wear rate threshold of 0.05 mm/year eliminates the risk of osteolysis. The wear for HXLPE is reported to be substantially lower, down to 0.002 mm/year (Thomas et al. 2011, Snir et al. 2014, Glyn-Jones et al. 2015). Its improved wear resistance is related to the different manufacturing process of the liners; by different amount of radiation, annealing, or remelting of the polyethylene; and even different sterilizing techniques. To date, there is no clear evidence for superiority regarding wear for any of the manufacturing processes. Even when fundamental wear improvements occur, clinical effects require many years before being obvious, thus strengthening the importance of conducting long-term clinical studies as well as involving different processing techniques and manufacturers. Although wear of conventional PE and HXLPE has previously been compared in several studies, indicating superiority of HXLPE, there are to our knowledge only 2 comparable long-term prospective RSA studies (Johanson et al. 2012, Glyn-Jones et al. 2015). These studies, however, evaluate not only products from other manufacturers but different cross-linking processes as well. Furthermore, it is still debatable when the initial deformation (creep phase) ends, and when the actual wear phase begins for the different types of polyethylene. RSA is a reliable, validated method of assessing wear (Stilling et al. 2012).

The CSF cup with standard conventional PE liner (JRI Orthopaedics Ltd, London, UK) has been on the market since 1991 showing satisfactory results (Datir and Angus 2010, Raman et al. 2012). The CSF Plus cup (JRI Orthopaedics Ltd, London, UK) was introduced in 2006, as an evolution of the CSF cup, with a slightly rougher and improved surface in combination with a new HXLPE liner. We measured and compared the possible differences between the 2 generations of this manufacturer’s polyethylene liners in terms of creep, wear, cup migration, and clinical outcome up to 10 years. Our hypothesis was that HXLPE would result in less wear and that the rougher cup surface would yield better cup stability. Additionally, the patients were evaluated with the clinical Hip Disability and Osteoarthritis Outcome Score (HOOS) throughout the follow-up period.

## Patients and methods

### Study group

This is a single-center prospective cohort study conducted at Skåne University Hospital of 50 patients who had surgery performed between April 2007 and June 2008. Mean age was 63 years (50–75), 25 were men, all had primary hip OA, Charnley class A or B (Table 1), and had been included in a published randomized controlled trial comparing 2 versions of the Furlong stem (Weber et al. 2014).

**Table 1.** Patient characteristics

	CSF	CSF Plus	Total
	n = 23	n = 22	n = 45
Mean age (range)	64 (50–74)	62 (53–75)	63 (50–75)
Male/female sex	14/9	11/11	25/20
Mean BMI (SD)	27 (4.1)	29 (6.0)	28 (5.1)

SD = standard deviation.

**Figure 1.**
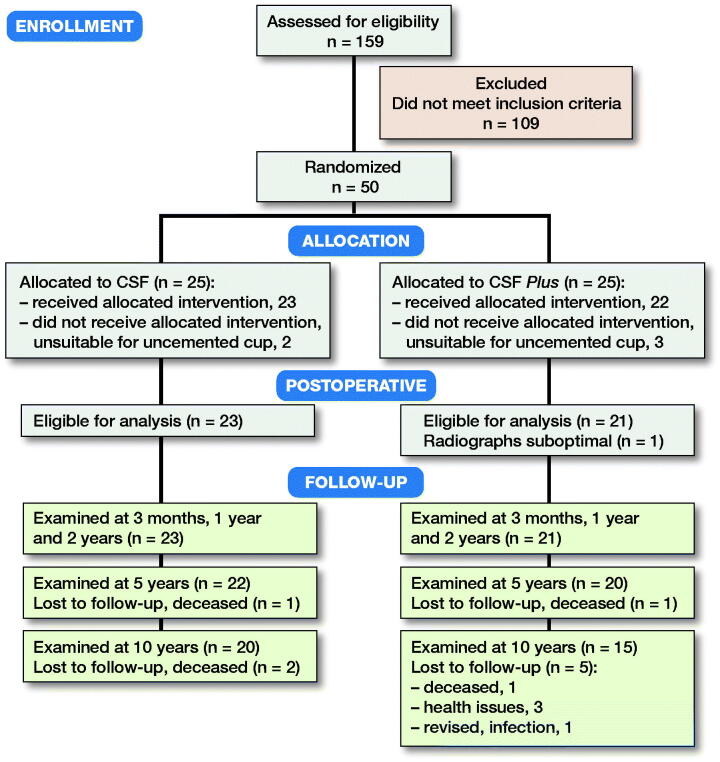
Consort flow chart.

Of the 50 patients, the first 25 were allocated to have a CSF cup with conventional PE liner and the following 25 patients a CSF Plus cup with HXLPE liner. The reason for this consecutive allocation on the cup side is that the CSF Plus cup was not available to us when the study was initiated but was obtainable later in the study period. Although all patients met the inclusion criteria and were suitable for an uncemented stem, 5 were considered unsuitable for an uncemented cup (women ≥ 70 years old with radiographical doubt as to bone quality in the acetabulum). Thus, 2 patients in the CSF group, and 3 in the CSF Plus group were excluded from the cup part of the study (Figure 1, Table 1).

In the cup migration analysis part of the study, due to the absence of adequate visible markers in the acetabulum affecting RSA cup migration measurements but not wear, 5 patients were excluded: 1 from the CSF group, 4 from the CSF Plus group. Liner analysis was conducted in all 35 patients.

### Surgery

Surgery was performed by 2 experienced hip surgeons (GF and MS) using a posterolateral incision. The patients were blocked randomized to have either the Furlong HAC or the Furlong Active stem (Weber et al. 2014). All patients received a 28 mm CoCr head (JRI Orthopaedics Ltd, London, UK). The cup liners were marked by the surgeons by insertion of 4–6 tantalum markers (dependent on liner size) using drilled holes in standardized positions (0.8 mm diameter) in the periphery of the liner. Additionally, 6–9 Tantalum markers were placed in the periacetabular bone in the pelvis.

The liner in the CSF cup is made from Ticona grade GUR 1050 resin, which is ram extruded and sterilized with 2.5 Mrads. The HXLPE liner in the CSF Plus cup is made from the same material followed by irradiation with 7.5 Mrads of gamma sterilization to produce the cross-linking in the polyethylene. The liners are free from calcium stearate, a compound that has been associated with fusion defects and increased oxidation (McKellop et al. 1999). The polyethylene is then remelted to closely restore its mechanical properties. The product is finally sterilized with 2.5–4 Mrads in vacuum.

The CSF Plus cup metal shell, compared with its precursor CSF, has a thicker and rougher layer of titanium coating. The complete coating includes the same outer layer of hydroxyapatite, Supravit, 100–170 µ thick for both cups. This makes the total thickness for CSF Plus 365 µ with a roughness of 60–100 RZ, compared with a total thickness of 200 µ and a roughness of 30–50 RZ for the CSF cup shell.

### RSA

RSA examinations were performed according to the guidelines for standardization for radiostereometry (Valstar et al. 2005). The reference RSA examination was performed on the 1st postoperative day, before full weight-bearing, and then at 3, 12, 24, 60, and 120 months, with a time interval of ±5% for each follow-up examination.

During the follow-up period, all patients had a double examination calculating the precision value (Table 2).

**Table 2.** Precision values of the cup wear and migration analysis

	Liner wear	Cup	Cup
Axis	translation (mm)	translation (mm)	rotation (°)
Transverse (X)	–	0.14	0.76
Longitudinal (Y)	0.08	0.09	0.72
Sagittal (Z)	–	0.38	0.22
3D	0.23	–	–

The value given represents the smallest migration considered as statistically significant and is based on mean + 2 SD of the error obtained. This corresponds to the 95% confidence limit.

An upper limit for the condition number (CN) is normally set at 150 (Valstar et al. 2005). We had a mean of 25 for all examinations and none of the accepted exceeding 125. The upper limit for mean error of rigid body fitting (ME) was set at 0.30 with a mean for all examinations of 0.05. The RSA examinations were performed using a uniplanar technique with the patient in a supine position and the calibration cage below the patient (Selvik 1989, Kärrholm et al. 1997). We used UmRSA software for the analysis (version 6.0; RSA Biomedical, Umeå, Sweden) and a type 41 calibration cage (Tilly Medical AB, Lund, Sweden).

Point motion of the femoral head in relation to the cup segment was used for wear analysis. The cup segment was defined as cup opening and back shell as definitive points of the cup combined with the markers from the liner periphery (Börlin et al. 2006). The femoral head penetration into the cup liner could be measured along the 3 axes in an orthogonal coordinate system, as signed values of X-, Y-, and Z-translation, as well as total penetration as the total point motion (3D vector). Proximal head penetration (Y-translation) and 3D penetration were selected as primary effect variables as these are the most representative of the wear direction. For cup migration analysis, segment motion of the cup segment was compared in relation to the pelvis segment. The proximal migration (Y-translation) and change of inclination (Z-rotation) were chosen as primary effect variables for cup migration with the others as secondary.

Cup inclination was measured for all patients on the first postoperative radiographs as this can affect the wear of the liner (Tian et al. 2017).

### Clinical assessment

The Self-administered Hip Disability and Osteoarthritis Outcome Score (HOOS) (Nilsdotter et al. 2003) was filled out by all patients before surgery and at 12, 24, 60, and 120 months.

### Statistics

Power analysis was performed based on previously published RSA data on stems and cups. Assuming that the true difference of head penetration at 2 years is 0.1 mm with a common standard deviation (SD) of 0.1, 21 patients in each group would yield a power of 90% to find a statistically significant difference between the groups, using alpha = 0.05. To cover possible dropouts, 25 patients were included in each group.

Continuous variables are presented using mean and SD or range, and categorical variables are presented using counts and percentages. A significance level of 0.05 was used for all statistical tests and 95% confidence intervals (CI). Comparison between CSF and CSF Plus at single time-points were performed using two-sample t-tests. Linear regression was used to evaluate the effect of cup slope on wear.

Wear over time was analyzed using a piecewise linear mixed-effect model with a knot (breaking point) at 3 months after surgery where a clear pattern change from creep to deformation has been shown in an earlier study (Bergvinsson et al. 2020). The models included 3 fixed effects: group, time starting from surgery, and time starting from 3 months after surgery, and 2 interaction terms between group and the time variables. Subject was included as a random effect. These models gave the opportunity to compare the wear slopes before and after the breaking point between the 2 cup types. Before performing the actual analyses, data was reviewed to confirm the assumption that the breaking point is at 3 months after surgery.

The HOOS data was analyzed using Mann–Whitney U-test for comparison between groups.

### Ethics, funding, and potential conflicts of interests

The study was approved by the Ethics Committee of Lund University, Sweden (Dnr 2007/33). All patients gave informed written consent to participate in the study including follow-ups. The study was carried out according to the Helsinki Declaration of 1975, as revised in 2000. Data is available on reasonable request.

JRI Orthopaedics Ltd have financially supported part of the RSA examinations but had no influence on how this study was conducted or how the results were interpreted. The authors have no conflict of interest.

## Results

### RSA

**Figure 2.**
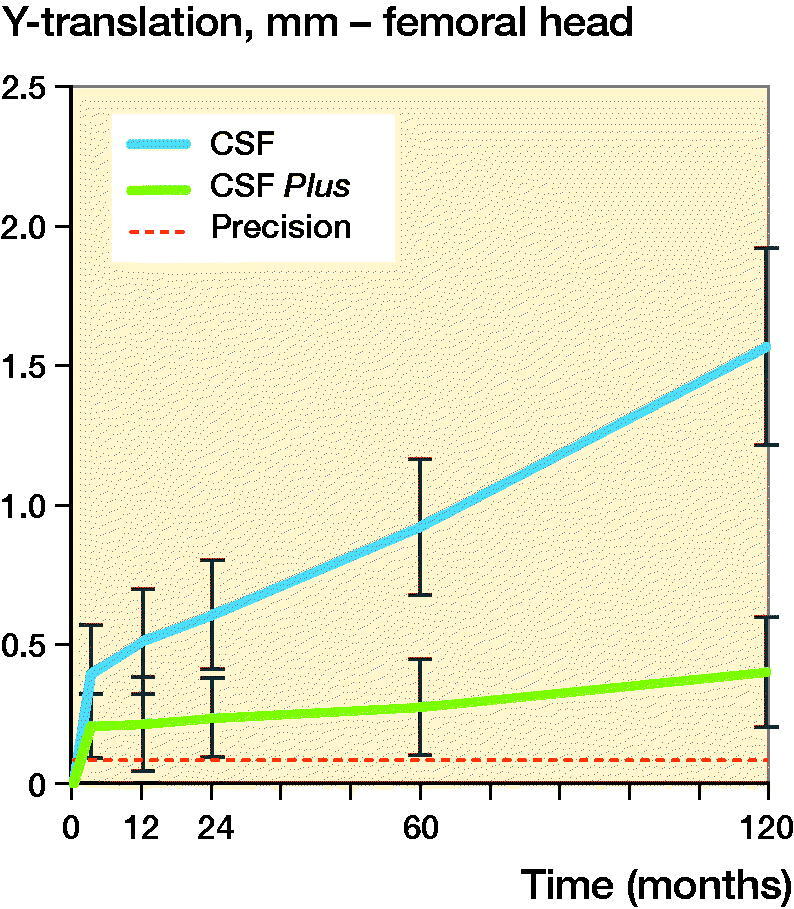
Y-translation of the femoral head for conventional PE (CSF) and HXLPE (CSF Plus) with 95% CI bars.

Both groups showed head penetration into the liner occurring during the first 3 months, known as initial polyethylene deformation or creep. The mean Y-penetration at 3 months was 0.39 (CI 0.21–0.60) mm for the conventional PE group and 0.21 (CI 0.10–0.32) mm for the HXLPE group. After this there is a clear change in the wear pattern, indicating change from the initial deformation phase followed by beginning of the wear phase. Based on this observation, a mixed–model analysis was performed with a knot at the 3-month follow-up moment. Between 3 months and 10 years the mean femoral head penetration in the 2 groups showed different patterns. The head penetration in the conventional PE group continued (p < 0.001, mixed models) whilst the HXLPE group experienced minimal penetration (p = 0.3, mixed models); at 10 years the total Y-translation was 1.56 (CI 1.21–1.91) mm and 0.40 (CI 0.20–0.60) mm, respectively. This results in a yearly wear rate of 0.12 mm for conventional PE and 0.02 mm for HXLPE after the initial creep period (Table 3 and Figure 2).

**Table 3.** Wear measured with RSA as translation of femoral head. Values are mean (mm) and (95% confidence intervals)

Months	Conventional PE	HXLPE	**p-value** a
Y-axis translation			
3	0.39 (0.22–0.57)	0.21 (0.09; 0.32)	0.07
12	0.51 (0.32–0.70)	0.21 (0.04–0.38)	
24	0.60 (0.42–0.80)	0.24 (0.09–0.38)	
60	0.92 (0.68–1.16)	0.27 (0.10–0.45)	< 0.01
120	1.56 (1.21–1.92)	0.40 (0.20–0.60)	
3D translation			
3	0.62 (0.38–0.87)	0.40 (0.26–0.55)	0.09
12	0.71 (0.47–0.96)	0.50 (0.32–0.67)	
24	0.81 (019–1.04)	0.50 (0.33–0.67)	
60	1.12 (0.86–1.37)	0.54 (0.38–0.70)	< 0.01
120	1.69 (1.30–2.08)	0.56 (0.31–0.81)	

a Mixed models analysis between 0 and 3 months and 3 months to 10 years, respectively

**Figure 3.**
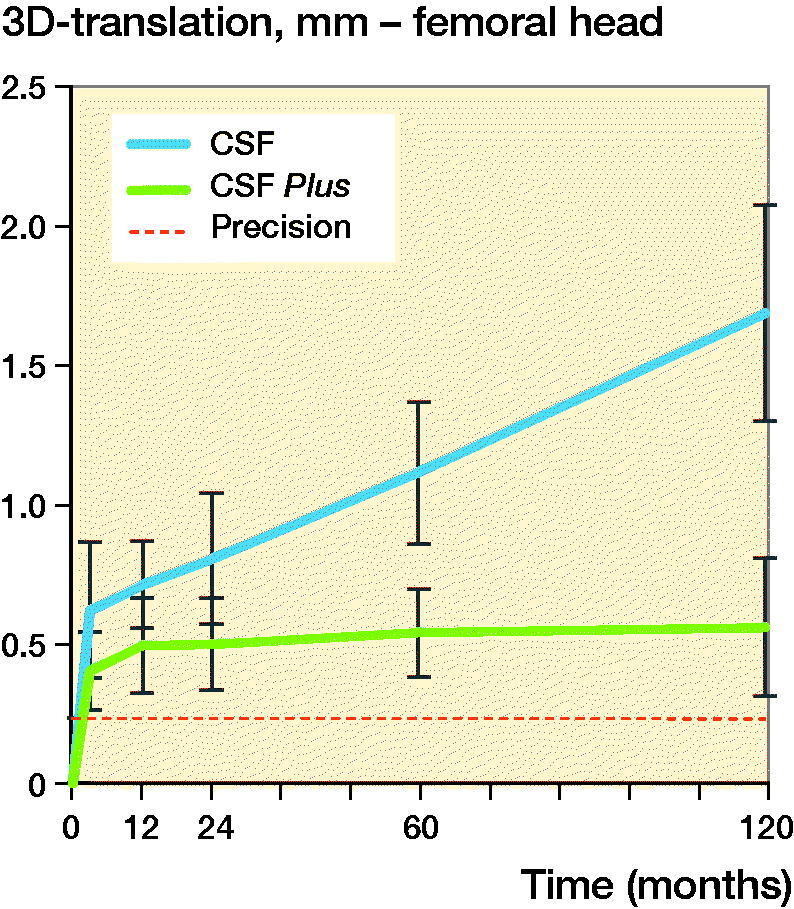
3D-translation of the femoral head for conventional PE (CSF) and HXLPE (CSF Plus) with 95% CI bars.

The values for the total penetration (3D vector) were similar’ at 3 months, the wear was 0.62 (CI 0.37–0.87) mm for the conventional PE group and 0.40 (CI 0.26–0.54) mm for the HXLPE group. The total wear, at 10 years, for the conventional PE group was 1.69 (CI 1.30–2.08) mm and 0.56 (CI 0.31–0.81) mm for the HXLPE group. Thus, for the conventional PE the yearly wear rate is 0.11 mm/year compared with 0.02 mm/year for the HXLPE group (Table 3 and Figure 3).

**Figure 4.**
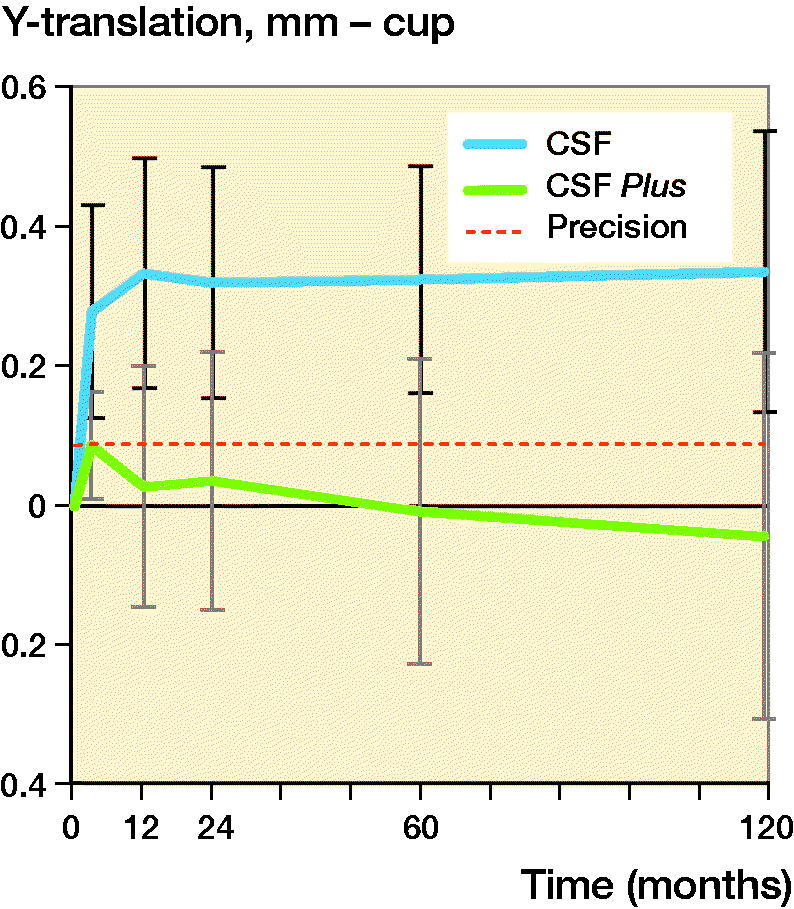
Y-translation of the CSF and CSF Plus cups with 95% CI bars.

**Figure 5.**
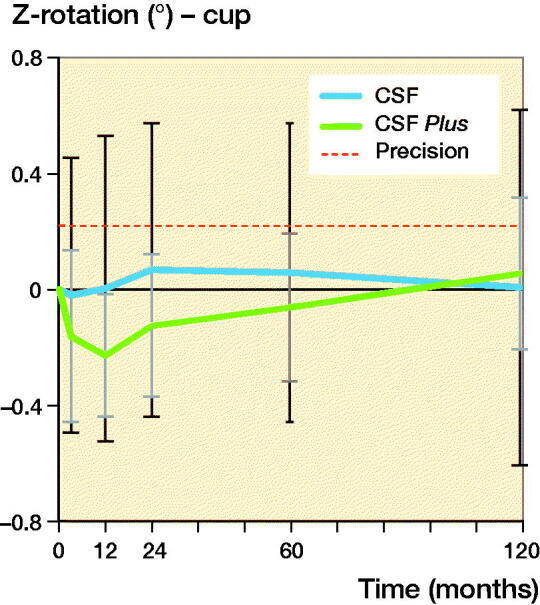
Z-rotation of the CSF and CSF Plus cups with 95% CI bars. Plus (+) rotation indicates decreased and minus (–) rotation increased inclination.

The CSF cup in the conventional group migrated cranially (Y-translation) 0.28 mm (CI 0.13–0.43) during the first 3 months and then seemed to have stabilized with a migration of 0.34 mm at 10 years. The CSF Plus cups in the HXLPE group had a Y-translation from 0.09 (CI 0.01–0.17) mm at 3 months and –0.04 (CI –0.30 to 0.22) mm at 10 years. After initial settling-in, measured up to 3 months, there was, up to 10 years, generally very little translation and rotation of the cups in both groups (Figures 4 and 5 and Table 4).

**Table 4.** Cup migration. Values are mean (mm/°) and (95% confidence intervals)

Months	CSF	CSF Plus
X–axis translation, medial (+) or lateral (–)		
3	0.37 (0.16 to 0.59)	0.17 (–0.06 to 0.40)
12	0.35 (0.12 to 0.57)	0.17 (–0.05 to 0.40)
24	0.37 (0.14 to 0.59)	0.25 (–0.01 to 0.50)
60	0.31 (0.04 to 0.58)	0.31 (0.03 to 0.60)
120	0.31 (0.02 to 0.60)	0.30 (0.04 to 0.56)
Y–axis translation, proximal (+) or distal (–)		
3	0.28 (0.13 to 0.43)	0.09 (0.01 to 0.16)
12	0.34 (0.17 to 0.50)	0.03 (–0.15 to 0.20)
24	032 (0.16 to 0.49)	0.04 (–0.15 to 0.22)
60	0.33 (0.16 to 0.49)	–0.01 (–0.23 to 0.21)
120	0.34 (0.13 to 0.54)	–0.04 (–0.31 to 0.22)
Z–axis translation, anterior (+) or posterior (–)		
3	0.03 (–0.15 to 0.21)	0.19 (–0.08 to 0.46)
12	–0.01 (–0.20 to 0.17)	0.35 (–0.03 to 0.74)
24	0.10 (–0.09 to 0.30)	0.38 (–0.04 to 0.80)
60	0.11 (–0.09 to 0.32)	0.13 (–0.27 to 0.53)
120	0.04 (–0.21 to 0.28)	0.23 (–0.27 to 0.73)
X–axis rotation, anterior (+) or posterior (–) tilt		
3	0.26 (–0.20 to 0.71)	0.10 (–0.13 to 0.33)
12	0.40 (–0.01 to 0.80)	0.21 (–0.24 to 0.67)
24	0.28 (–0.14 to 0.71)	0.08 (–0.43 to 0.58)
60	0.25 (–0.26 to 0.77)	0.17 (–0.37 to 0.72)
120	0.20 (–0.34 to 0.74)	0.23 (–0.60 to 1.06)
Y–axis rotation, internal (+) or external (–) rotation		
3	–0.15 (–0.60 to 0.29)	0.05 (–0.33 to 0.44)
12	–0.11 (–0.49 to 0.27)	0.00 (–0.42 to 0.42)
24	–0.16 (–0.57 to 0.25)	–0.12 (–0.57 to 0.33)
60	–0.13 (–0.51 to 0.25)	–0.29 (–0.77 to 0.20)
120	–0.03 (–0.56 to 0.51)	–0.27 (–0.76 to 0.22)
Z–axis rotation, decreased (+) or increased (–) inclination		
3	–0.02 (–0.49 to 0.46)	–0.16 (–0.46 to 0.14)
12	0.00 (–0.52 to 0.53)	–0.23 (–0.44 to –0.02)
24	0.07 (–0.44 to 0.58)	–0.12 (–0.37 to 0.12)
60	0.06 (–0.46 to 0.58)	–0.06 (–0.32 to 0.19)
120	0.01 (–0.61 to 0.62)	0.06 (–0.21 to 0.32)

### Radiography

The mean cup inclination, as measured on the postoperative radiographs, was 43° (CI 41–46) for the conventional PE group and 44° (CI 42–46) for the HXLPE group.

### Clinical outcome (HOOS)

**Figure 6.**
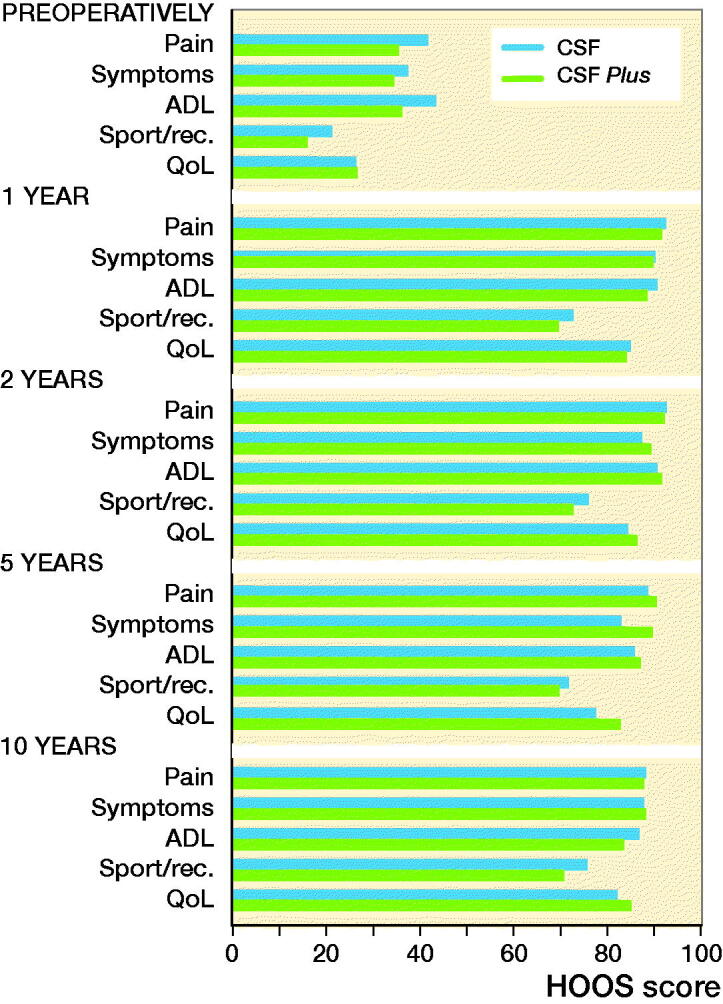
HOOS questionnaire outcome. HOOS outcome measures: Pain; Symptoms including stiffness and range of motion; Activity limitations – daily living (ADL); Sport and recreation function (Sport/Rec.); and Hip-related quality of life (QoL). A score of 0 indicates poor function/high number of symptoms, a score of 100 indicates excellent function/low number of symptoms.

The HOOS was similar for both groups preoperatively and at 6, 12, 24, 60, and 120 months. All patients had improved HOOS scores compared with preoperatively and the improvement remained up to 10 years (Figure 6).

1 cup was revised due to late hematogenic infection. 10 years after THA, none of the remaining cups had any clinical or radiological signs of loosening requiring revision.

## Discussion

This study was conducted in order to investigate the long-term difference between conventional PE and HXLPE. Our results confirms that the superiority of the HXLPE continues up to 10 years. The curves indicate that this pattern will continue and, so far, we cannot see any disadvantages with the change from conventional PE to HXLPE. Furthermore, we conclude that the deformation process of the PE liner can be divided into 2 phases: the initial deformation phase, known as creep (Sychterz et al. 1999) and the later true wear phase of the liner. It has been proposed that most of the early deformation occurs within the 1st postoperative year (Sychterz et al. 1999, Hopper et al. 2004). Our results indicate that most of the creep has already happened within the first 3 months and after this initial phase the wear pattern changes from creep to actual wear. However, this is probably a gradual and overlapping process, which we speculate ends within the 1st year. Based on our results we have chosen to calculate wear rate from 3 months onwards. The wear of the conventional PE is steady during the whole study period from 3 months to 10 years with annual wear rate of 0.12 mm/year. The HXLPE show less initial creep and then also exhibits a steady wear pattern but remarkably less compared with the conventional PE, with mean annual wear rate of only 0.02 mm/year. Thus, the HXLPE is less prone to wear than its precursor and has a wear rate well below the threshold limit for wear induced osteolysis of 0.1 mm/y (Dowd et al. 2000, Dumbleton et al. 2002). However, the older conventional PE can be at risk. Our results are consistent with previously published studies indicating that the conventional PE has a higher wear rate than HXLPE, while the latter has a wear rate ranging from 0.002 to 0.15 mm/year and continues to be low even at long-term follow up (Engh et al. 2012, Reynolds et al. 2012, Babovic and Trousdale 2013, Glyn-Jones et al. 2015, Teeter et al. 2017, Tsukamoto et al. 2017). Our 10-year follow-up RSA data indicates a lower risk of later osteolysis and aseptic loosening for HXLPE.

There are several ways of producing the HXLPE: by different radiation intensity, annealing or remelting, and sterilizing techniques, resulting in variations in characteristics of the liners. Although the superiority of each technique is debatable, studies to date indicate no increased risk in use of HXLPE compared with their precursors. To our knowledge, this is the 1st study presenting wear data on this specific manufacturer’s HXLPE.

Our secondary aim was to investigate possible difference in migration behavior as well as time required until osseointegration for the CSF and the CSF Plus cup occurred. The newer design, with a rougher surface, showed less migration during initial bedding-in. However, both seemed to have osseointegrated within 3 months, and none of them showed further signs of migration and/or associated loosening throughout the 10-year follow-up. This is considered a good migration pattern for acetabular cups and indicates minimal risk for aseptic loosening with revision risk in the long term (Pijls et al. 2012).

The mean pain score in HOOS was 92 (100 being no pain) for both groups 1 year after surgery and 88 after 10 years. Hence, patients experienced their hips still performing well after 10 years.

A limitation of the study is that this is not a randomized study for the cup part, but only for the stem part of the study. Instead, the patients were operated on consecutively with the 1st half of the patients receiving the CSF cup and conventional PE liner and the other half receiving CSF Plus cups and HXLPE liner. The reason for this was that the CSF Plus cups were not released when the study started. It should be noted that there was the same proportion of the different stems in each group. Another potential limitation is that we are comparing the 2 different kinds of polyethylene in 2 slightly different cup shells. It might be speculated that the HXLPE liner is somewhat affected by the slightly rougher surface of the CSF Plus shell compared with the conventional PE of the CSF shell. However, we find this unlikely as the migration behavior of the cups was very similar, except for a slightly less early migration of the CSF Plus cup. A further potential limitation is that due to loss to follow-up after 10 years, the remaining 20 and 15 patients in each respective group do not meet our initial criteria for power in the study. However, the differences in wear already at 5 years are far greater than the values used for power calculations, leading us to believe that these results have sufficient power.

In conclusion, our results indicate that this HXLPE has the wear characteristics expected from a modern HXLPE, with markedly less wear compared with the older conventional PE. Both the older cup, with conventional PE, and the newer cup with its slightly rougher surface and an HXLPE liner indicate very good stability up to 10 years.

HB: study conduction, data analysis, writing of the manuscript. GF and MS: study design and conduct, performing surgery, data analysis, and critical revision of the manuscript. VZ: critical revision of the manuscript.

The authors would like to thank Håkan Leijon at the RSA laboratory, Skåne University Hospital, Lund University for computerizing and analyzing the RSA pictures; Helene Jacobsson at Clinical Studies Sweden—Forum South, Skåne University Hospital, Lund is thanked for statistical guidance.  

*Acta* thanks Lennard Koster and Matthew Teeter for help with peer review of this study.
